# Targeted vs. systematic early antiviral treatment against A(H1N1)v influenza with neuraminidase inhibitors in patients with influenza-like symptoms: Clinical and economic impact

**DOI:** 10.1371/currents.RRN1121

**Published:** 2009-10-27

**Authors:** Sylvie Deuffic-Burban, Xavier Lenne, Benoit Dervaux, Julien Poissy, Xavier Lemaire, Caroline Sloan, Fabrice Carrat, Jean-Claude Desenclos, Jean-Francois Delfraissy, Yazdan Yazdanpanah

**Affiliations:** ^*^INSERM U795 and EA2694, Faculté de Médecine de Lille, Lille, France; ^†^CRESGE-LEM UMR 8179, Université Catholique de Lille, Lille, France; ^‡^EA2694, Faculté de Médecine de Lille, Lille, France; ^§^Service Universitaire des Maladies Infectieuses et du Voyageur, Centre Hospitalier Dron, Tourcoing, France; ^¶^Service Universitaire des Maladies Infectieuses et du Voyageur, Centre Hospitalier Dron, Tourcoing, France; Service de Medecine Interne, Centre Hospitalier de Douai, France; ^#^INSERM UMR-S 707, UPMC, Paris, France; Unité de Santé Publique, AP-HP, Hôpital Saint-Antoine F-75012; ^**^Institut de Veille Sanitaire (InVS), Saint Maurice, France; ^††^Service de Médecine Interne et des Maladies Infectieuses, AP-HP, Hôpital Bicêtre, Le Kremlin-Bicêtre, France and ^‡‡^EA2694, Faculté de Médecine de Lille, Lille, France; Service Universitaire des Maladies Infectieuses et du Voyageur, Centre Hospitalier Dron, Tourcoing, France

## Abstract

Capitalizing on available data, we used a decision model to estimate the clinical and economic outcomes associated with early initiation of treatment with neuraminidase inhibitors in all patients with influenza-like illnesses ( ILI ) (systematic strategy) vs. only those at high risk of complications (targeted strategy). Systematic treatment of ILI during an A(H1N1)v influenza epidemic wave is both effective and cost-effective. Patients who present to care with ILI during an A(H1N1)v influenza epidemic wave should initiate treatment with neuraminidase inhibitors, regardless of risk status. Administering neuraminidase inhibitors between epidemic waves, when the probability of influenza is low, is less effective and cost-effective.

## **Introduction**

On April 29 2009, the World Health Organization (WHO) announced that the A(H1N1) virus (A(H1N1)*v*), which is composed of a unique combination of gene segments never before observed in human or swine influenza viruses, was spreading rapidly across the globe [1]. Since April, A(H1N1)*v* influenza has spread from an initial outbreak in Mexico and the southern United States to a total of 150 countries in October 2009 [2] [3] [4] [5] [6] [7]. Of the roughly 375,000 laboratory-confirmed cases of A(H1N1)*v* influenza worldwide, over 4,500 were fatal [8].

A(H1N1)*v* can be treated with neuraminidase inhibitors such as oseltamivir and zanamivir. The virus is susceptible to these drugs in the first 48 hours of infection. In all high-income countries except for the United Kingdom, early antiviral treatment with neuraminidase inhibitors is only recommended for A(H1N1)*v*-infected patients who have severe symptoms or underlying conditions associated with a high risk of developing seasonal influenza complications [9] [10] [11] [12] [13]. These recommendations are based on our current understanding of seasonal human influenza. However, recent data have shown that, unlike with seasonal influenza [14], a high proportion of severe and fatal A(H1N1)*v* complications occur in previously healthy young people [15] [16] [17] [18] [19]. At the National Institute of Respiratory Diseases in Mexico City for instance, of the first 18 patients who were hospitalized for pneumonia and laboratory-confirmed A(H1N1)*v *influenza, only eight had preexisting medical conditions [16]. Similarly, of the first 32 patients who were admitted to intensive care units (ICU) with A(H1N1)*v *influenza in Spain, 15 had no preexisting medical conditions [18]. In a recent analysis that used complementary data from two cities in the United States, Presanis *et al*. found a higher symptomatic case-fatality ratio in the non-elderly adult group than in the elderly group (0.136% 18-64 years *vs*. 0.028% >65 years) [17]. Given these data and the decreased efficacy of neuraminidase inhibitors >48 hours after the onset of symptoms [20] [21], policy makers have to decide whether to recommend treating all patients who present to care with influenza-like illnesses (ILI), or just those at high risk of influenza complications.

Several prevention and treatment options for A(H1N1)*v* influenza are currently available. However, the data needed to make an informed decision are incomplete and results of clinical trials and/or epidemiological cohort studies will not be available in time to curb this rapidly spreading disease. Decision-analysis methods provide a systematic approach to synthesizing existing data and quantifying the trade-offs for alternative options. Capitalizing on available data to date, we used a decision model to estimate during an epidemic wave the clinical and economic outcomes associated with early initiation of antiviral treatment with neuraminidase inhibitors in all symptomatic patients *vs*. only those at high risk of influenza complications.

## **Material and methods**

### Study design

We built a decision model to compare two antiviral treatment strategies of A(H1N1)*v* influenza management during an epidemic wave in France: early treatment with neuraminidase inhibitors in patients who present to care with ILI and are at high risk of complications, as currently recommended in France (targeted strategy) [12], *vs*. early treatment with neuraminidase inhibitors in all patients who present to care with ILI (systematic strategy).

We evaluated each strategy in a cohort of 64,300,000 people, the size of the French population in 2009. The incidence of the simulated disease follows the trajectory of a unique pandemic A(H1N1)*v* influenza wave. Model outcomes include mortality, overall hospitalizations, ICU admissions, cost, and cost-effectiveness. Costs consisted only of direct medical costs. Cost-effectiveness is defined as the additional cost of one strategy compared to another strategy divided by its additional clinical benefit [22] [23]. Outcomes were assessed from the French health care provider perspective. Clinical benefits, expressed in years of life gained (YLG), were discounted at 3% per year. Costs, expressed in 2009 euros (€1.00 = US$1.48 = £0.91 on 24 September 2009) were not discounted, because all costs are incurred in the first year.

### Model structure (Figure 1)

The root node of the decision tree divides into two branches that represent each treatment strategy. Patients who present with ILI can be at high or low risk of developing complications from A(H1N1)*v*. Patients can follow various pathways, depending on the probability of the following events: (i) development of symptomatic ILI; (ii) presentation to care given symptomatic ILI; (iii) A(H1N1)*v* influenza given presentation to care with ILI; (iv) hospitalization given presentation to care and diagnosis of A(H1N1)*v*; (v) ICU admission given hospitalization; and (vi) death given ICU admission. 

In the targeted strategy, patients with ILI initiate antiviral treatment if they are hospitalized or if they are at high risk of complications. Transition probabilities for these patients were derived from the A(H1N1)*v* influenza epidemic in New Zealand, where targeted antiviral treatment is currently recommended [10]. These probabilities therefore incorporate the efficacy of antiviral treatment in high-risk patients. In the systematic strategy, patients who present to care with ILI <48 hours after the onset of symptoms and are at low risk of complications also receive treatment. We assume that a proportion of these patients present to care >48 hours after the onset of symptoms and do not benefit from antiviral treatment. Treatment efficacy is defined as the percent reduction in hospitalizations among patients with ILI who present to care <48 hours after the onset of symptoms. In both strategies, patients initiate oseltamivir treatment without confirmatory testing for A(H1N1)*v* influenza. The mean daily dose of oseltamivir is 30 mg x2 for infants aged <one year, 45mg x2 for children aged 1-12 years, and 75 mg x2 for children and adults aged >12 years.

### Input data

According to French recommendations on antiviral treatment use in patients with A(H1N1)*v* influenza, high-risk patients include: patients aged >65 years; infants aged <one year; pregnant women; and patients with chronic respiratory diseases, chronic cardiovascular diseases, diabetes, obesity and immunosuppression [24]. The elderly and infant population in France represents 17.8% of the overall population [25]. The number of pregnant women at time *t* was estimated from the average number of births in France over 9 months in 2008, and represents 0.9% of the French population [25]. We estimated the number of patients aged <65 years who suffer from chronic respiratory disease, chronic cardiovascular disease, diabetes, obesity and immunosuppression from data on chronic disease treatment reimbursements in the French national health system. These patients represent 3% of the French population [26]. Overall, in a population of mean age 40 years, 23% of people were estimated to be at high risk of developing influenza complications.

The characteristics and outcomes of the A(H1N1)*v* influenza epidemic in the targeted strategy are largely based on data from the 11-week A(H1N1)*v* influenza epidemic in New Zealand, where the influenza season is nearly over (Table 1) [27]. In New Zealand, 34.9% of the overall population developed ILI, 5.5% of symptomatic people presented to care, and 21.5% of patients who presented to care were diagnosed with A(H1N1)*v* influenza. The attack rate was 7.5%. Among patients who presented to care with A(H1N1)*v* influenza, 5.5% were hospitalized. Using data from Germany, we estimated that high-risk patients were 2.7 times more likely to be hospitalized than low-risk patients [15]. In a recent analysis, Jain *et al*. did not find an increased risk of ICU admission or death in patients with underlying medical conditions [19]. Therefore, the probability of being admitted to the ICU given hospitalization (12%) and the probability of death given ICU admission (14%) was assumed to be the same in both low- and high-risk groups. We varied all of these values in the sensitivity analysis.

When low-risk patients with A(H1N1)*v* influenza presented to care <48 hours after the onset of symptoms, antiviral treatment reduced the probability of hospitalization by 60%, as reported by Kaiser *et al*. [28]. We estimated the proportion of low-risk patients who present to care <48 hours after the onset of symptoms from an analysis of the first A(H1N1)*v* influenza cases in Germany [15]. This study showed that the average time between onset of symptoms and antiviral treatment initiation in 1,810 patients was 2.2 days, with a decreasing trend from week 28 (4.0 days) to week 32 (2.0 days) of the analysis. We assumed that 25% of low-risk patients who presented to care with ILI started treatment on the first day of symptoms and 25% started treatment on the second day. The last 50% of patients presented to care too late to benefit from antiviral treatment. Furthermore, some studies have suggested that the beginning of symptoms is difficult to recall accurately in 49% of cases of influenza because of the insidious onset of the disease [29] [30]. We assumed that 49% of patients who initiated treatment on the second day did not recall the onset of their symptoms accurately, and that half of them presented to care >48 hours after the onset of symptoms. The proportion of patients who presented to care and initiated treatment <48 hours after the onset of symptoms was thus 43.9% ([25% + 25%] - [25% x 50% x 49%]).

Clinic visit costs are from the French Nomenclature Générale des Actes Professionels [31] and drug costs are from national medication rates [32]. The cost of a primary care clinic visit was €21 and five-day oseltamivir treatment costs ranged from €13 to €25, depending on dosage. We derived inpatient costs for ILI from 2006 French hospital information system (Programme de Médicalisation des Systèmes d’Information) data [33] [34], which differentiate ICU admissions from regular inpatient visits [35] [36]. We considered costs for all patients assigned to diagnostic-related group (DRG) 04M13Z (pulmonary edema and respiratory distress) and international classification of diseases (ICD)-10 category J96.0 (acute respiratory failure) [37]. The average length of inpatient stay was four days and was derived from the French A(H1N1)*v* influenza surveillance system, which was set up in the spring of 2009 [38].

### Sensitivity analysis

In univariate sensitivity analysis, we varied key parameters over a wide range of reasonablevalues to evaluate the impact of data uncertainties on our overall conclusions. First, we varied the mean age of the A(H1N1)*v*-infected population, the proportion of patients at high risk of influenza complications, and the A(H1N1)*v* attack rate. Varying the probability of presentation to care with ILI without varying the probability of A(H1N1)*v* influenza given ILI allowed us to vary the attack rate. Second, we varied the probability of presentation to care with ILI in both strategies to evaluate the impact of higher overall rates of presentation to care in France compared to New Zealand. We also increased this probability among low-risk patients in the systematic strategy without changing the targeted strategy, to test the hypothesis that universal antiviral use might encourage patients to seek care. In this scenario, we assumed that none of the additional patients developed complications or required medical care. Third, we lowered the probability of hospitalization, increased the difference in ICU admission rates between low- and high-risk patients, and varied the rates and duration of hospitalizations and ICU admissions. Finally, we varied the proportion of patients initiating antiviral treatment <48 hours after the onset of symptoms, as well as oseltamivir efficacy (Table 1).

We conducted two two-way sensitivity analyses. We performed the first to investigate the impact of differences in resource utilization among patients with ILI in France and in New Zealand. In this sensitivity analysis, we increased the probability of presentation to care with ILI and decreased the probability of hospitalization given presentation to care. According to available data on resource utilization related to seasonal influenza [39] [40] [41] [42], patients with ILI present to care more frequently in France, but probably their symptoms are less severe than in New Zealand. Thus the probability of hospitalization given presentation to care with ILI is lower in France than in New Zealand [39] [40] [41] [42]. In the second two-way sensitivity analysis, we varied oseltamivir efficacy and the probability of A(H1N1)*v* influenza given presentation to care with ILI across broad ranges, In these two-way sensitivity analyses we generally considered scenarios that reduced the benefits of the systematic strategy.

### Software

The model was built with TreeAge ProTM 2006 software (Williamstown, MA,USA).

## **Results**

### Base-case analysis (Table 2)

When the targeted strategy was implemented over the course of an 11-week A(H1N1)*v* influenza epidemic, 14,460 patients were hospitalized, 1,696 were admitted to the ICU, and 238 died. In the systematic strategy, 12,339 patients were hospitalized, 1,447 were admitted to the ICU, and 203 died. Compared to the targeted strategy, the systematic strategy thus reduced hospitalizations by 2,121, ICU admissions by 249, and deaths by 35. Overall direct medical costs were €86,237,980 in the targeted strategy and €89,591,030 in the systematic strategy. The systematic strategy increased life expectancy by 817 discounted life years for an additional cost of €3,353,050 compared to the targeted strategy, leading to a cost-effectiveness ratio of €4,100/YLG.

### Sensitivity analyses (Table 3)

In univariate sensitivity analysis, results were most sensitive to variations in the probability of A(H1N1)*v* influenza given presentation to care with ILI, the probability of hospitalization given diagnosis with A(H1N1)*v* influenza, and oseltamivir efficacy. When we reduced the probability of diagnosing A(H1N1)*v* influenza in patients who present to care with ILI from 21.5% to 5%, systematic treatment reduced deaths by eight and led to a cost-effectiveness ratio of €49,300/YLG compared to the targeted strategy. When we decreased the probability of hospitalization from 5.5% to 1.37% in patients with A(H1N1)*v* influenza, systematic treatment reduced deaths by eight and produced a cost-effectiveness ratio of €45,500/YLG compared to the targeted strategy. When oseltamivir reduced the probability of hospitalization by 5% rather than 60% in the base case, the systematic strategy reduced deaths by three and was associated with a cost-effectiveness ratio of €155,200/YLG.

Variations in the epidemic attack rate had an impact on overall deaths and costs, but not on cost-effectiveness. When we increased the attack rate from 7.5% to 20%, reductions in mortality produced by systematic treatment increased from 35 to 93. Overall costs increased from €3,353,050 to €8,941,480. 

Increases in the probability of presentation to care with ILI among low-risk patients had an impact on cost-effectiveness in univariate sensitivity analysis. When we doubled the proportion of low-risk patients who presented to care in the systematic strategy and assumed that 50% of them received antiviral treatment, the cost-effectiveness of systematic treatment compared to targeted treatment was €40,000/YLG. Cost-effectiveness ratios remained stable when we increased the probability of presentation to care with ILI in both low- and high-risk patients. When we simultaneously increased presentation to care with ILI and decreased hospitalizations, cost-effectiveness ratios increased. For example, when we increased the probability of presentation to care to 30% and decreased the probability of hospitalization to 1%, the systematic strategy led to a cost-effectiveness ratio of €65,900/YLG compared to the targeted strategy.

In a second two-way sensitivity analysis, we varied both oseltamivir efficacy and the probability of diagnosing A(H1N1)*v* influenza in patients who present to care with ILI (Figure 2). Under the conservative assumption that antiviral treatment reduces the probability of hospitalization by 10% and that 15% of patients with ILI are A(H1N1)*v*-infected, the cost-effectiveness of systematic treatment compared to targeted treatment increased to €108,200/YLG. When we further reduced the proportion of A(H1N1)*v*-infected patients to 10% and 5%, the systematic strategy led to cost-effectiveness ratios of €167,200/YLG and €344,000/YLG compared to the targeted strategy. 

## **Discussion**

We used available data on the A(H1N1)*v* influenza epidemic in New Zealand to build a decision model and assess the clinical benefits and cost-effectiveness of different antiviral treatment strategies in the French population. Systematic initiation of neuraminidase inhibitors in patients who present to care with ILI during an A(H1N1)*v* influenza epidemic wave reduces mortality compared to a strategy that only targets patients at high risk of developing complications, and leads to a cost-effectiveness ratio of €4,100/YLG. According to the World Health Organization Commission on Macroeconomics and Health, an intervention is cost-effective if it delivers life years at a cost below three times the per capita Gross Domestic Product (GDP) (€91,240 for France in 2008) [43]. The systematic strategy falls well below this threshold. Clinical benefits and cost-effectiveness were sensitive to the probability of A(H1N1)*v* influenza in patients who present to care with ILI, the probability of hospitalization among A(H1N1)*v*-infected patients and, most importantly, oseltamivir efficacy. Clinical benefits were also sensitive to variations in the A(H1N1)*v *influenza attack rate and the probability of ICU admission given hospitalization. 

To assess the internal validity of the model, we compared the model-based incidence of ICU admissions to the incidence of ICU admissions recently reported in Australia and New Zealand. The model projected 26.4 admissions per million people, compared to 28.7 (95% confidence interval [CI], 26.5-30) in Australia and New Zealand [44]. Model projections were within 10% and almost within the 95% CI of reported ICU admissions.

To our knowledge, the cost-effectiveness of antiviral treatment against A(H1N1)*v* influenza has never been evaluated, although several studies have investigated the impact of treating patients with seasonal human influenza [29] [45] [46] [47] [48]. The cost-effectiveness of antiviral treatment in these studies varies widely [49], particularly when treatment is compared to supportive care. These differences are mainly due to variations in study perspective, cohort characteristics, and other model parameters. Although study results should be compared with caution, it should be noted that the cost-effectiveness of systematic treatment in our study is lower than in previous studies, despite the absence of quality of life considerations in our analysis. In a recent study on seasonal human influenza, the probability that patients who presented to care with ILI had influenza was 46% and the probability of presentation to care <48 hours after the onset of symptoms was 20%. The authors found that oseltamivir treatment in low-risk persons aged 12-65 years was associated with a cost-effectiveness ratio of £32,400 per quality-adjusted life year saved (QALY; 2009 €36,600/QALY) compared to no treatment [29]. The cost-effectiveness ratios reported in these studies are higher than in our study, most likely because the hospitalization and mortality rates associated with seasonal influenza among young and low-risk populations are lower than for A(H1N1)*v* influenza [14] [15] [16] [17] [18]. The severity of the current A(H1N1)*v *pandemic is uncertain because data on prevalence and hospitalization rates are imperfect. We addressed this issue by using data from New Zealand, where the influenza season is nearly over, and by performing a wide range of sensitivity analyses, particularly on the probability of hospitalization among patients who present to care with A(H1N1)*v* influenza, as reported by Presanis *et al*. in the United States [17]. Systematic treatment with neuraminidase inhibitors in patients who present to care with ILI remained effective and cost-effective in sensitivity analysis.

Variations in antiviral treatment efficacy had a large impact on results. When we decreased the efficacy of oseltamivir, the clinical benefits of systematic treatment decreased and cost-effectiveness ratios increased. A recent systematic review and meta-analysis has suggested that, even for seasonal human influenza, data are insufficient to draw conclusions on the impact of neuraminidase inhibitors on complication rates in low- and high-risk populations [50]. Our estimate of oseltamivir efficacy is from Kaiser *et al*.’s meta-analysis of 10 placebo-controlled double-blind trials of oseltamivir treatment, which enrolled a total of 3,564 subjects with ILI. This meta-analysis found however very few events in both low- and high-risk populations [28]. A study conducted with data from Thailand’s National Avian Influenza Surveillance (NAIS) system showed that survival after treatment with oseltamivir was associated with a crude odds ratio of 0.11 (95% CI, 0.04-0.30) [51]. This estimate is higher than ours, but it was derived from a retrospective observational study and should therefore be considered with caution. A recent study performed in the United States suggests that all patients with A(H1N1)*v* influenza benefit from antiviral treatment, regardless of risk status. Among patients who were hospitalized, were not admitted to the ICU, and survived, 45% initiated antiviral treatment <48 hours after the onset of symptoms. Among patients who were hospitalized, were admitted to the ICU, and died, only 23% initiated antiviral therapy early [19]. These data are consistent with our hypothesis on antiviral treatment efficacy. However, we varied oseltamivir efficacy over a wide range in sensitivity analysis. When oseltamivir reduced hospitalization rates by <8%, the clinical benefits of systematic treatment were low and the cost-effectiveness ratio increased to above the WHO threshold for cost-effectiveness [43].

Results were sensitive to the probability of diagnosing A(H1N1)*v* influenza in patients with ILI. When this probability decreased, the systematic strategy became less effective and less cost-effective. When we set it to 5% and decreased oseltamivir efficacy, clinical benefits were small and cost-effectiveness ratios increased sharply. Given uncertainties surrounding the efficacy of neuraminidase inhibitors, and in the absence of rapid A(H1N1)*v* diagnostic tests that might reduce inappropriate neuraminidase inhibitor use, physicians should initiate antiviral treatment in all patients with ILI when A(H1N1)*v* reaches epidemic rates, but not between epidemic waves.

Mortality rates were especially sensitive to the A(H1N1)*v* influenza attack rate. When we increased the attack rate, mortality differences between the systematic strategy and the targeted strategy increased, but cost-effectiveness ratios remained stable. The attack rate in our analysis, derived from New Zealand data, is lower than predictions for upcoming A(H1N1)*v* epidemics. If A(H1N1)*v* influenza attack rates increase, the systematic strategy will be much more effective and cost-effectiveness ratios will remain stable. 

This study has several limitations. First, our model combines input data from several sources and relies on several assumptions to estimate the impact of different antiviral treatment strategies. In particular, we used data from New Zealand on the probabilities of presenting to care with ILI, A(H1N1)*v* influenza given presentation to care with ILI, hospitalization given A(H1N1)*v*, ICU admission given hospitalization, and death given ICU admission [27]. Although prevention and control measures, and recommendations for A(H1N1)*v* pandemic influenza management and antiviral treatment use in New Zealand are similar in many other developed countries [9] [10] [11] [12], the characteristics of the epidemic and healthcare resource utilization may be different in other settings. However, our results remained stable when we used more conservative estimates for the probabilities of presentation to care with ILI and hospitalization given ILI. Second, the impact of neuraminidase inhibitors on complication rates is uncertain. Our results were robust to variations in treatment efficacy, except when the probability of diagnosing A(H1N1)*v* influenza in patients with ILI was low. Third, we did not account for oseltamivir-related adverse events, but these events are rare and it is unlikely that they would impact our results. Fourth, we did not account for resistance to oseltamivir, which could become more frequent as the availability of neuraminidase inhibitors increases. However, resistance generally emerges in patients who take antiviral drugs as pre- or post-exposure prophylaxis, or because of severe immunosuppression [52] [53] [54]. Fifth, we did not consider the impact of A(H1N1)*v* influenza on quality of life or productivity and the impact of antiviral treatment on influenza transmission. In addition, we did not incorporate the economic benefits of stockpiling oseltamivir in preparation for an eventual influenza pandemic, as has been done in France and other countries. Because antiviral treatment is already in stock, medical costs in France will be lower than those used in our study. The systematic strategy is even more cost-effective if we take these parameters into account. Finally, we did not consider the effects of A(H1N1)*v* influenza vaccination. The results of our study cannot be extrapolated to a vaccinated population because the severity of disease in vaccinated and unvaccinated populations may differ. However, it is likely that a large proportion of the general population in France will refuse to be vaccinated [55], and most countries have recommended vaccination for high-risk groups only [56] [57].

Guidelines for antiviral treatment use in patients with A(H1N1)*v* influenza are currently based on our understanding of seasonal human influenza. However, the proportion of healthy young people with A(H1N1)*v* influenza who develop severe or fatal complications is higher than for seasonal human influenza. As demonstrated in this analysis, systematic treatment of all patients with ILI during an A(H1N1)*v *influenza epidemic wave is both effective and cost-effective. Additional data on the efficacy of neuraminidase inhibitors in preventing influenza complications should be collected to improve clinical and cost-effectiveness estimates. Meanwhile, all patients who present to care with ILI during an A(H1N1 influenza epidemic wave should initiate treatment with neuraminidase inhibitors. Decision makers and clinicians should use caution when administering neuraminidase inhibitors between epidemic waves, when the probability of influenza is low.

## **Tables and Figures**

### **Table 1. Summary of input parameters for** **a decision model of A(H1N1)*v* influenza treatment**

**Table:** 

**Variable**	**Baseline value**	**Range**	**Source**
Cohort characteristics Mean age, years Life expectancy, years Proportion of patients at high risk of complications, %^*^	64,300,000 40 41 23	- 20 60 23-40	[25]
A(H1N1)*v* epidemic characteristics in the targeted strategy			
Attack rate, %	7.5	7.5-15.0	[27]
Probability of ILI, %^†^	34.9	34.9-69.8	[27]
Probability of presenting to care with ILI, %^‡^	5.5	5.5-30.0	[27]
Probability of A(H1N1)*v* given presentation to care with ILI, %^§^	21.5	5.0-15.0	[27]
Probability of hospitalization given presentation to care with ILI and A(H1N1)*v*, %^║^	5.5	1.4-5.5	[17] [27]
Increased hospitalizations in high-risk patients *vs.* low-risk patients ^¶^	2.7	-	[15]
Probability of ICU admission given hospitalization, %^**^	12	6-24	[27]
Increased ICU admissions in high-risk patients *vs.* low-risk patients	1.0	1.0-3.0	[27]
Probability of death given ICU admission, %^**^	14	-	[27]
Antiviral treatment use and efficacy in the systematic strategy			
Proportion of patients who present to care and initiate treatment <48 hours after the onset of symptoms, %^††^	50	25-75	[15] [29] [30]
Proportion of patients who initiate treatment >48 hours after the onset of symptoms, %	6.1	3.1-9.2	[15] [29] [30]
Antiviral treatment efficacy, %	60	5-30	[28]
Health care resource utilization and costs	** **	** **	** **
Clinic visit	21	-	
Average length of inpatient stay, days	4	7	[38]
Average length of ICU admission, days	13	21	[38]
Cost per inpatient day, 2009 €	482€	-	[35] [36] [37]
Cost per ICU day, 2009 €	1,319€	-	[35] [36] [37]
5-day oseltamivir treatment by age, 2009 € <1 year 1-12 years >12 years	12.69 18.78 24.85	- - -	[32]
ILI: influenza-like illness; ICU: intensive care unit ^*^Patients at high risk of complications include patients aged >65 years; infants aged <1 year; pregnant women; and patients with chronic respiratory diseases, chronic cardiovascular diseases, diabetes, obesity and immunosuppression. ^†^The probability of ILI is 0.019062 x 18.3, from Baker *at al*. [27]. In this study, only one in 18.3 patients with ILI presented to care and the cumulative rate of presentation to care with ILI during an epidemic wave was 1.9062%. ^‡^The probability of presenting to care with ILI is 1/18.3, from Baker *et al*. [27]. ^§^The probability of A(H1N1)*v* given presentation to care with ILI is 408.9/1906.2, from Baker *et al*. [27]. In this sudy, the cumulative rate of A(H1N1)*v* diagnosis among patients presenting to care with ILI during an epidemic wave was 408.9/100,000. ^║^The probability of hospitalization given presentation to care with ILI and A(H1N1)*v* diagnosis is 972/17672, from Baker *et al*. [27]. ^¶^The difference in hospitalization rates between low- and high-risk patients is (20/108)/(220/3162), from Gilsdorf *et al*. [15]. In this study, the hospitalization rate was 20/108 in high-risk patients and 220/3162 in low-risk patients. ^**^The probability of ICU admission given hospitalization is from Baker *et al*. [27]. In this study, 114/972 hospitalized patients were admitted to the ICU, and 16/114 ICU admissions died. ^††^Based on the physician’s deduction.			

### **Table 2. Base case results for different antiviral treatment strategies in France**

**Table:** 

	**Hospitalizations**		**Deaths**	**Discounted life years**	Undiscountedcosts (€)^*^	**CER (€/LYG)**
	**Overall**	**ICU**				
Targeted strategy^†^	14,460	1,696	238	5,573	86,237,980	
Systematic strategy^‡^	12,339	1,447	203	4,755	89,591,030	4,100
ICU: intensive care unit; CER: cost-effectiveness ratio; LYG: life years gained ^*^Costs were not discounted, because all costs were incurred within 1 year of presentation to care. ^†^Early treatment with neuraminidase inhibitors in patients who present to care with ILI and are at high risk of complications, as currently recommended in France. ^‡^Early treatment with neuraminidase inhibitors in all patients who present to care with ILI.						

### **Table 3. Sensitivity analyses**

**Table:** 

** **	**Targeted strategy**			**Systematic strategy**			**CER**
** **	**Deaths**	**Discounted life years**	Cost (€)^*^	**Deaths**	**Discounted life years**	Cost (€)^*^	**€/LYG**
Base-case	238	5,573	86,237,980	203	4,755	89,591,030	4,100
Mean age (base case: 40 years)							
20 years	238	6,587	86,237,980	203	5,521	89,591,030	2,500
Percent of patients at high risk of complication (base case: 23%)							
30%	238	5,573	88,311,420	209	4,887	91,915,250	5,300
40%	238	5,573	91,973,470	215	5,044	94,927,350	6,900
Attack rate (base case: 7.5%)							
15%	476	11,145	172,475,960	406	9,511	179,182,060	4,100
20%	635	14,860	229,967,940	542	12,680	238,909,420	4,100
Probability of presenting to care with ILI (base case: 5.5%)							
10%	436	10,198	157,815,500	372	8,702	163,951,590	4,100
20%	871	20,396	315,631,000	743	17,405	327,903,180	4,100
Probability of A(H1N1)*v* influenza given presentation to care with ILI (base case: 21.5%)							
15%	166	3,897	70,092,030	142	3,325	75,812,970	10,000
10%	111	2,598	57,577,840	95	2,217	65,134,040	19,800
5%	55	1,299	45,063,640	47	1,108	54,455,120	49,300
Probability of hospitalization given presentation to care with ILI and A(H1N1)*v* (base case: 5.5%)							
1.37%	59	1,388	45,922,220	51	1,184	55,187,790	45,500
Probability of presenting to care with ILI (base case: 5.5%) and probability of hospitalization given presentation to care with ILI and A(H1N1)*v* (base case: 5.5%)							
10% and 3.0%	238	5,562	113,154,200	203	4,747	125,840,090	15,600
20% and 1.5%	238	5,562	172,719,690	203	4,747	205,950,530	40,700
30% and 1.0%	238	5,562	232,285,170	203	4,747	286,060,970	65,900
Probability of ICU admission given hospitalization (base case: 12%)							
6%	122	2,851	73,631,840	104	2,433	78,833,650	12,400
24%	487	11,403	113,243,380	416	9,731	112,635,950	Cost-saving
Increased ICU admissions in high-risk *vs*. low-risk patients (base case: 1)							
Í2	279	6,537	90,705,530	251	5,873	94,766,380	6,100
Í3	307	7,198	93,765,500	284	6,638	98,311,140	8,100
Proportion of patients who present to care ≤48 hours after the onset of symptoms (base case: 50%)^‡^							
25%	238	5,573	86,237,980	221	5,164	87,914,510	4,100
75%	238	5,573	86,237,980	186	4,347	91,267,560	4,100
Proportion of new patients treated when proportion of low-risk patients presenting to care with ILI is doubled (base case: 5.5%)^†^							
0	238	5,573	86,237,980	203	4,755	108,325,390	27,000
50%	238	5,573	86,237,980	203	4,755	118,938,660	40,000
Antiviral treatment efficacy (base case: 60% reduction in hospitalization rate)							
30%	238	5,573	86,237,980	221	5,164	93,527,880	17,800
15%	238	5,573	86,237,980	229	5,368	95,496,300	45,300
5%	238	5,573	86,237,980	235	5,505	96,808,590	155,200
Average length of inpatient stay (base case: 4 days)							
7 days	238	5,573	104,694,610	203	4,755	105,340,900	800
Average length of ICU admission (base case: 13 days)							
21 days	238	5,573	104,133,170	203	4,755	104,861,910	900
Discount rate (base case: 3%)							
0%	238	9,759	86,237,980	203	8,328	89,591,030	2,300
5%	238	4,116	86,237,980	203	3,513	89,591,030	5,600
ICU: intensive care unit; CER: cost-effectiveness ratio; LYG: life years gained ^*^Costs were not discounted, because all costs were incurred within 1 year of presentation to care. ^†^We only varied this probability in low-risk patients in the systematic strategy, to test the hypothesis that universal antiviral use might encourage these patients to seek care. ^‡^We assumed that 49% of patients who initiated treatment on the second day of symptoms did not recall the onset of their symptoms accurately, and that half of them presented to care >48 hours after the onset of symptoms [29] [30]. In sensitivity analysis, the proportion of patients who presented to care and initiated treatment <48 hours after the onset of symptoms was reduced by (25% or 75%) x 50% x 49%.							

### **Figure 1**

Decision tree. Targeted strategy: early treatment with neuraminidase inhibitors in patients who present to care with ILI and are at high risk of complications, as currently recommended in France. Systematic strategy: early treatment with neuraminidase inhibitors in all patients who present to care with ILI. Patients at high risk of complications include patients aged >65 years; infants aged <1 year; pregnant women; and patients with chronic respiratory diseases, chronic cardiovascular diseases, diabetes, obesity and immunosuppression.

\begin{equation*}\circ \end{equation*} decision node  \begin{equation*}\diamond \end{equation*} probability node \begin{equation*}\triangle \end{equation*} end of one cycle

### **Figure 2**

Impact of variations in antiviral treatment efficacy and the probability of A(H1N1)*v* influenza in patients who present to care with influenza-like illnesses on the cost-effectiveness of systematic treatment compared to targeted treatment. Antiviral efficacy was defined as the % reduction in hospitalization rates. The horizontal line represents 3 x the annual per capita gross domestic product (GDP) for France (= €91,240 in 2008). The World Health Organization (WHO) Commission on Macroeconomics and Health guidelines [43] considers interventions below this threshold to be cost-effective.

**Figure:**
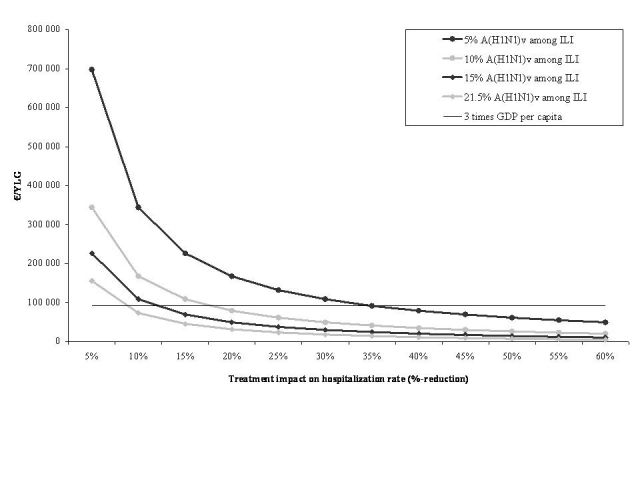

## **Acknowledgments**

The authors gratefully acknowledge the Institut de Microbiologie et Maladies Infectieuses for its support.

### Funding information

 This study received financial support from the Institut de Microbiologie et Maladies Infectieuses.

### Competing interests

S. Deuffic-Burban has received grants from Roche and Janssen-Cilag. F. Carrat acts as consultant for and has received grants from Roche, GlaxoSmithKline, Sanofi-Aventis, and Novartis and has been paid to attend meetings. Y. Yazdanpanah has received travel grants, honoraria for presentation at workshops and consultancy honoraria from Bristol-Myers Squibb, Gilead, Glaxo-SmithKline, Merck, Pfizer, Roche and Tibotec. None of the other authors report any association that might pose a conflict of interest.
